# GUT MICROBIOTA ALTERATIONS IN RODENT MODELS OF CHOLESTASIS INDUCED BY BILE DUCT LIGATION: A SYSTEMATIC REVIEW

**DOI:** 10.1590/S0004-2803.24612025-159

**Published:** 2026-07-24

**Authors:** Stephane Lima CALIXTO, Ana Caroline Lopes de Paula MACEDO, Jair Adriano Kopke AGUIAR

**Affiliations:** 1 Universidade Federal de Juiz de Fora, Instituto de Ciências Biológicas, Departamento de Bioquímica, Laboratório de Análise de Glicoconjugados, Juiz de Fora, MG, Brasil.; 2 Universidade Federal de Juiz de Fora, Centro de Bioterismo Regional, Juiz de Fora, MG, Brasil..

**Keywords:** Cholestasis, gut microbiota, bile duct ligation, animal models, Colestase, microbiota intestinal, ligadura do ducto biliar, modelos animais

## Abstract

**Background and objective:**

Cholestatic liver diseases are a major public health issue, marked by impaired bile flow and significant disruptions in liver and systemic physiology. Growing evidence points to the gut microbiota as a key player in cholestasis pathogenesis through gut-liver axis interactions. This systematic review aimed to synthesize and evaluate current findings on intestinal microbiota changes in rodents (rats and mice) subjected to bile duct ligation (BDL)-induced cholestasis, focusing on microbial diversity, taxonomic shifts, and potential pathophysiological implications.

**Methods:**

A comprehensive literature search was conducted in PubMed, Scopus, and Embase for studies published from January 2020 to February 2025, following PRISMA guidelines. Eligible studies included original research using BDL in rodents without therapeutic intervention and reporting gut microbiota profiles. Data were qualitatively analyzed, emphasizing experimental conditions and microbiome outcomes.

**Results:**

Twenty-two studies met inclusion criteria. Most used 16S rRNA sequencing; two used shotgun metagenomics. BDL consistently induced gut dysbiosis, with reductions in alpha diversity (in most studies), altered beta diversity, and shifts in dominant phyla such as *Firmicutes*, *Bacteroidetes*, *Proteobacteria*, *Actinobacteria*, and *Verrucomicrobiota*. At finer taxonomic levels, increases in *Prevotella*, *Enterococcus*, *Escherichia coli*, and *Alistipes* were common, while *Lactobacillus* and *Ruminococcus* often decreased. Elevated levels of *Akkermansia muciniphila* and *Bifidobacterium pseudolongum* may represent compensatory microbial responses.

**Conclusion:**

Bile duct ligation (BDL)-induced cholestasis leads to complex changes in the microbiota that can worsen intestinal barrier integrity, increase bacterial translocation, and intensify liver inflammation. These findings reinforce the central role of the gut-liver axis and corroborate the potential of microbiota-targeted therapies in the management of cholestatic liver diseases. However, as most of the available evidence derives from experimental models, further well-designed clinical studies are needed to validate the safety, efficacy, and translational applicability of these strategies in human diseases.

## HIGHLIGHTS

Cholestatic liver diseases involve gut-liver axis disruption, and this review summarizes evidence on gut microbiota alterations in rodents with BDL-induced cholestasis.

A PRISMA-based systematic search (2020-2025) of PubMed, Scopus, and Embase included only studies using BDL without therapeutic interventions.

Evidence consistently shows BDL-induced dysbiosis, with reduced diversity and significant taxonomic shifts, including increased opportunistic taxa and reduced beneficial genera.

These alterations may impair intestinal barrier integrity, enhance bacterial translocation, and exacerbate liver inflammation, supporting microbiota-targeted therapeutic approaches.

## RESUMO

**Contexto e objetivo:**

As doenças hepáticas colestáticas são um importante problema de saúde pública, caracterizadas por fluxo biliar prejudicado e alterações significativas na fisiologia hepática e sistêmica. Evidências crescentes apontam a microbiota intestinal como um fator-chave na patogênese da colestase por meio de interações no eixo intestino-fígado. Esta revisão sistemática teve como objetivo sintetizar e avaliar as descobertas atuais sobre as alterações da microbiota intestinal em roedores (ratos e camundongos) submetidos à colestase induzida por ligadura do ducto biliar (BDL), com foco na diversidade microbiana, alterações taxonômicas e potenciais implicações fisiopatológicas.

**Métodos:**

Uma busca abrangente na literatura foi realizada no PubMed, Scopus e Embase para estudos publicados de janeiro de 2020 a fevereiro de 2025, seguindo as diretrizes PRISMA. Os estudos elegíveis incluíram pesquisas originais utilizando BDL em roedores sem intervenção terapêutica e que relatassem perfis da microbiota intestinal. Os dados foram analisados qualitativamente, com ênfase nas condições experimentais e nos resultados do microbioma.

**Resultados:**

Vinte e dois estudos atenderam aos critérios de inclusão. A maioria utilizou sequenciamento de rRNA 16S; dois estudos utilizaram metagenômica shotgun. A ligadura do ducto biliar (BDL) induziu consistentemente disbiose intestinal, com reduções na diversidade alfa (na maioria dos estudos), alteração na diversidade beta e mudanças em filos dominantes, como Firmicutes, Bacteroidetes, Proteobacteria, Actinobacteria e Verrucomicrobiota. Em níveis taxonômicos mais refinados, aumentos em *Prevotella*, *Enterococcus*, *Escherichia coli* e *Alistipes* foram comuns, enquanto *Lactobacillus* e *Ruminococcus* frequentemente diminuíram. Níveis elevados de *Akkermansia muciniphila* e *Bifidobacterium pseudolongum* podem representar respostas microbianas compensatórias.

**Conclusão:**

A colestase induzida por BDL leva a alterações complexas na microbiota que podem piorar a integridade da barreira intestinal, aumentar a translocação bacteriana e alimentar a inflamação hepática. Esses achados reforçam o papel central do eixo intestino-fígado e corroboram o potencial de terapias direcionadas à microbiota no manejo de doenças hepáticas colestáticas. Contudo, como a maior parte das evidências disponíveis deriva de modelos experimentais, são necessários mais estudos clínicos bem delineados para validar a segurança, a eficácia e a aplicabilidade translacional dessas estratégias em doenças humanas.

## INTRODUCTION

In recent years, liver diseases have emerged as a significant challenge for public health systems worldwide, as their mortality rates have shown a steady increase since 1990. It is estimated that these conditions are responsible for approximately 2 million deaths annually, accounting for about 4% of all deaths recorded globally^1^ ^,^ ^2^. In addition to their impact on mortality, these diseases also impose a considerable economic burden due to the increase in hospital admissions, the need for diagnostic testing, the acquisition of pharmacological treatments, and, most notably, the growing demand for liver transplantation^3^.

Among liver diseases, cholestasis stands out as a clinically relevant condition, particularly due to its complex nature, which may involve immunological, genetic, and environmental components in its pathophysiology. It is also one of the leading causes of liver transplantation^4^ ^,^ ^5^. This condition is primarily characterized by the obstruction of bile flow, which impedes the proper transport of bile to the duodenum. Consequently, bile acids accumulate in the liver and reflux into the systemic circulation^4^. This dysfunction can trigger alterations in bile acid metabolism, as well as promote oxidative stress, inflammatory processes, and hepatic fibrosis, potentially progressing to cirrhosis and cancer^4^ ^-^ ^6^. Despite considerable research into the underlying mechanisms of cholestasis, the specific pathogenic pathways of cholestatic liver injury remain poorly understood, highlighting a significant gap in developing effective preventive strategies.

Recent evidence indicates that, in addition to the before mentioned pathophysiological changes, cholestasis is also associated with the occurrence of intestinal dysbiosis due to alterations in bile acid metabolism observed in affected patients^7^ ^,^ ^8^. The bidirectional interaction between bile acids and the intestinal microbiota plays a central role in maintaining intestinal homeostasis. The microbiota is essential for the proper metabolism of bile acids, which, in turn, possess cytotoxic properties capable of directly influencing the composition and bacterial load of the gut^9^ ^,^ ^10^. However, microbiota imbalance may compromise the integrity of the mucosal barrier, facilitating bacterial translocation, characterized by the entry of intestinal bacteria and their metabolites into the liver via the portal circulation. This process can trigger an inflammatory response, contributing to the progression of hepatic fibrosis^1^ ^,^ ^7^ ^,^ ^11^. A more profound elucidation of microbiota alterations associated with cholestatic liver disease may provide valuable insights into the pathophysiological mechanisms involved and guide the development of therapeutic strategies to mitigate dysbiosis and bacterial translocation, potentially slowing the progression of hepatic fibrosis.

Considering the persistent gaps in understanding the pathogenesis of cholestasis and the continued need for more effective therapeutic strategies, numerous studies have employed experimental animal models that reliably reproduce the clinicopathological features observed in humans^4^ ^,^ ^7^. Among these models, bile duct ligation (BDL) in rats and mice has become a well-established and widely used tool for studying cholestatic liver diseases^5^ ^,^ ^7^.

This model is based on a surgical procedure performed under anesthesia in which the bile duct is isolated, ligated, and transected. It is a highly reproducible technique characterized by the induction of acute hepatocellular injury and biliary infarcts. Moreover, it promotes a strong inflammatory response, as evidenced by immune cell infiltration, bile duct proliferation, and activation of myofibroblasts, which contribute significantly to the deposition of collagen and extracellular matrix in hepatic tissue^12^.

Given the above and the growing scientific interest in elucidating the interactions that constitute the gut-liver axis-particularly in the context of liver diseases-it is essential to systematically investigate how intestinal microbiota alterations manifest in experimental cholestasis models. Accordingly, the present study aims to comprehensively characterize the intestinal microbial profile in rodents, specifically rats and mice, subjected to cholestatic liver injury induced by the BDL technique, compared with control animals. To achieve this objective, a systematic review of the scientific literature published between January 2020 and February 2025 was conducted to identify recurring patterns, descriptive trends, convergences, and potential gaps in the current knowledge regarding microbiota alterations associated with this experimental model.

## METHODS

### Database and search strategy

This is a systematic review of the literature, developed following the PRISMA (Preferred Reporting Items for Systematic Reviews and Meta-Analyses) guidelines. The literature search was conducted using the following electronic databases: PubMed, Scopus, and Embase. The following descriptors and combinations were used with Boolean operators: (“Intestinal microbiota” OR “gut microbiota” OR “gut flora”) AND (“bile duct ligation” OR “bile duct obstruction” OR “cholestasis”) AND (“animal models” OR “rats” OR “mice”).

The search was limited to articles published between January 2020 and February 2025 in English, Portuguese or Spanish, with no restrictions regarding the country of origin.

### Eligibility criteria

The following inclusion criteria were used: 1) original articles involving the use of rats or mice; 2) cholestasis induced by the BDL model; 3) evaluation of the intestinal microbiota profile in both control animals and those subjected to the BDL model without any therapeutic intervention as one of the study outcomes; 4) publications from January 2020 to February 2025. And the exclusion criteria were as follows: 1) studies conducted in humans, other animal models, or in vitro; 2) reviews, editorials, conference abstracts, theses, or dissertations; 3) duplicate articles; 4) studies employing induction methods other than BDL; 5) studies that did not directly analyze the intestinal microbiota of control animals and those subjected to the BDL model without treatment; 6) studies with incomplete methodology or insufficient data for extraction.

### Study selection and data extraction

The eligible articles were assessed by two independent reviewers, and in cases of disagreement, a third reviewer was consulted. The following information was extracted from the included articles: article title and year of publication; species, strain, sex, age, and weight of the animals; electronic database used; experimental duration; methodology for microbiota analysis; main alterations in the intestinal microbial profile of control and untreated BDL groups.

The data were organized into tables and graphs generated using GraphPad Prism version 8.0 (GraphPad Software, San Diego, CA, USA) and Microsoft Excel and were analyzed descriptively and qualitatively following the aim of the present review.

## RESULTS AND DISCUSSION

**FIGURE 1:**
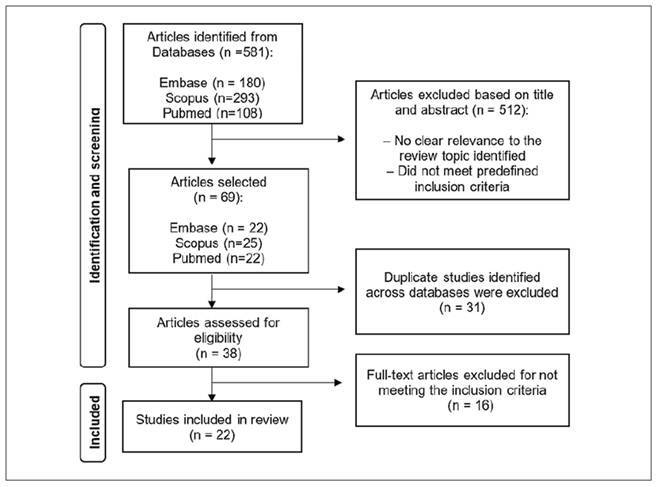
PRISMA flow diagram of the article selection process.

This systematic review included 22 studies published between 2020 and February 2025, which investigated the intestinal microbiota profile in rodents-rats and mice-subjected to the BDL technique without any therapeutic intervention compared to control animals. The PRISMA flow diagram illustrates the study selection process (Figure 1).

### Characterization of included studies by year of publication

**FIGURE 2:**
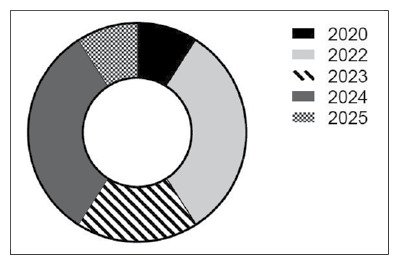
Characterization of the included studies by year of publication.

The analysis was based on articles published between January 2020 and February 2025. As shown in Figure 2, the years with the highest number of publications were 2022 and 2024, with 8 and 7 articles, respectively, followed by 2023 (4 articles), 2025 (2 articles), and 2020 (1 article). No studies meeting the predefined inclusion and exclusion criteria were identified for 2021.

It is worth noting that the years 2020 and 2021 coincided with the global health emergency caused by the novel coronavirus (COVID-19) pandemic, quickly becoming the third leading cause of mortality worldwide^13^. Given the magnitude of the health crisis, there was a significant reallocation of financial and human resources toward COVID-19-related research, resulting in a substantial increase in publications on this topic and, consequently, a reduction in publications in other areas, including liver diseases^14^ ^,^ ^15^. Furthermore, the sole study included from 2020 had already been accepted for publication in 2019, although it was officially published the following year. Thus, the scarcity of studies on hepatic cholestasis in 2020 and 2021 can plausibly be attributed to the redirection of scientific priorities during the pandemic.

Lastly, the low number of publications identified for 2025 may be explained by the fact that data collection was completed only through February.

### Characterization of Included studies regarding animal models

### Strains

**FIGURE 3:**
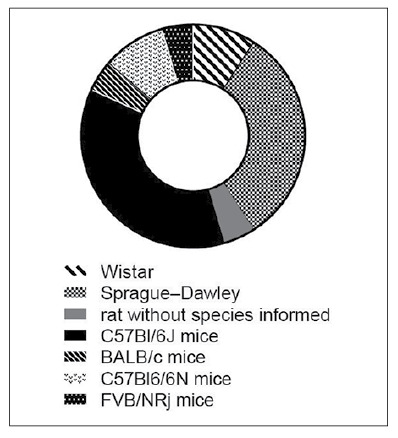
Characterization of the included studies according to the animal strains used.

Concerning the strains used in the analyzed studies (Figure 3), a diversity of rat and mouse strains was observed in this experimental model. The most frequently adopted strains were C57BL/6J mice, mentioned in 8 studies, and Sprague-Dawley rats, used in 7 studies. Other strains were reported less frequently, including Wistar rats (2 studies), C57BL/6N mice (2 studies), BALB/c mice (1 study), and FVB/NRj mice (1 study). Only one article did not specify the strain, mentioning only the use of rats.

The widespread use of C57BL/6J mice and Sprague-Dawley rats in BDL-induced hepatic cholestasis models is attributed to their well-established genetic, physiological, and practical characteristics in the scientific literature. C57BL/6J mice exhibit high genetic homogeneity, which minimizes variability between experimental groups and enhances the reproducibility of findings. Furthermore, this strain is highly responsive to immunological and metabolic stimuli and is widely employed in studies involving genetically modified animals, making it particularly suitable for mechanistic and molecular investigations^7^ ^,^ ^16^. Another important factor is their ability to develop systemic manifestations of advanced cholestasis, such as hyperammonemia and motor impairment-alterations not typically observed in other strains, such as Swiss mice. Additionally, C57BL/6J mice exhibit faster progression to cirrhosis following BDL-induced cholestasis, making them advantageous for studies focused on advanced stages of liver disease^17^. Sprague-Dawley rats, in turn, are notable for their body size, which is favorable for performing surgical procedures such as BDL, as well as for their docile temperament and ease of handling, which facilitates experimental management^16^.

Both strains display relevant pathophysiological responses that faithfully replicate clinical and histological aspects of human hepatic cholestasis, including fibrosis progression and hepatic inflammation^17^ ^,^ ^18^. Finally, the well-established use of these strains in biomedical research and the extensive documentation available in the literature further support their selection as reference models in cholestasis studies.

### Sex

**FIGURE 4:**
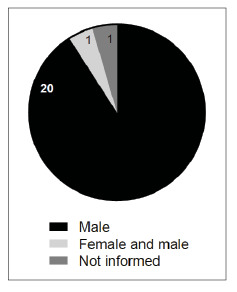
Characterization of the included studies according to the sex of the animals used.

Regarding the sex of the animals used, there was a marked predominance of male subjects, which were employed in 20 of the 22 included studies, as illustrated in Figure 4. Only one study reported using animals of both sexes, while another did not specify this information. The preference for male animals in these studies is grounded in physiological, hormonal, and methodological considerations.

Male animals exhibit lower hormonal variability than females, who undergo regular estrous cycles involving fluctuations in estrogen and progesterone levels-factors that can influence inflammatory and metabolic responses, compromising the consistency of experimental data. Additionally, estrogens exert hepatoprotective effects, which may reduce the severity of liver alterations induced by BDL, thereby complicating standardization between experimental groups^19^ ^,^ ^20^. Another determining factor is the larger body size of males, which facilitates the execution of invasive surgical procedures such as BDL, contributing to greater technical safety and protocol reproducibility.

### Weight and age

The selection criteria concerning the animals’ age and/or body weight revealed considerable heterogeneity among the studies, as detailed in Table 1. In studies that employed rats as the experimental model, animal selection was primarily based on body weight, ranging from 130 g to 400 g - an interval considered appropriate for performing invasive surgical procedures such as BDL.

**TABLE 1:** Characterization of the included studies according to the animals used, by weight and age.

Age or weight of animals	Animals species	Number of articles
130g	Sprague-Dawley	1
250-280g	Wistar	1
200-250g	Sprague-Dawley or Wistar	3
180-220g	Sprague-Dawley	1
20-24g	C57BL/6J	1
5-6 weeks (180-200g)	Sprague-Dawley	1
8 weeks (300-350g)	Sprague-Dawley	1
9 weeks (21.5-26.5g)	C57Bl6/6N	1
10 weeks (300-400g)	Sprague-Dawley	1
6 weeks	C57BL/6J	1
6-8 weeks	C57BL/6J	1
7 weeks	C57BL/6J	1
8 weeks	Balb/c or C57Bl6/6N	2
10 weeks	C57BL/6J	1
9-12 weeks	C57BL/6J	1
12 weeks	C57Bl/6J or	2
Not informed	FVB/NRj mice or Rats	2

In contrast, studies conducted with mice showed a predominant trend toward selection based on age, typically between 6 and 12 weeks, an ideal age range for ensuring the necessary physiological maturity without compromising surgical viability.

It is important to note that two studies did not provide precise information regarding the age or weight of the animals used. Nevertheless, analysis of the results indicates that, regardless of the age or weight of the selected animals, alterations in the microbiota of cholestatic animals induced by BDL were consistently observed.

### Characterization of the included studies according to experimental protocol duration

Concerning the duration of the experimental protocols, a wide variability was observed in the time intervals between the BDL surgery and the euthanasia of the animals for blood and tissue collection, as detailed in Table 2. This interval varied substantially among the analyzed studies, ranging from 48 hours to 30 days, reflecting distinct experimental objectives and methodological approaches. Among these, time points of 7, 14, and 21 days were the most frequently employed, each used in four different studies. The selection of these time frames is intrinsically related to the progression of the histopathological and physiological changes triggered by bile duct obstruction, which evolve in well-defined phases over the post-BDL period.

**TABLE 2:** Characterization of the included studies according to experimental protocol duration.

Experiment time	Number of articles
48 hours	1
3 days	1
5-7 days	1
7 days	4
11 days	1
12 days	1
14 days	4
15 days	1
21 days	4
26 days	1
30 days	2
Not informed	2

Although the temporal evolution of hepatic and systemic alterations is well documented, comparative analysis of the included studies did not reveal a consistent association between protocol duration and the patterns of intestinal microbiota alteration. Nevertheless, the data indicates that, regardless of the exposure duration following BDL-induced cholestasis, dysbiosis appears to be an early event with a tendency to persist or worsen over time.

### Characterization of the included studies regarding changes in intestinal microbiota composition

Most of the studies included in this review employed sequencing of the hypervariable regions of the 16S rRNA gene, allowing for the evaluation of relative abundance and bacterial composition across different taxonomic levels. This widely used approach in microbiome research provides good resolution for taxonomic analysis, particularly at the phylum and genus levels^21^.

Two studies opted for shotgun metagenomics, a more comprehensive technique that, in addition to taxonomic profiling, enables the identification of functional genes and metabolic pathways, thereby offering a more detailed view of the microbial alterations induced by cholestasis^22^ ^,^ ^23^.

In the present systematic review, the presentation of results and the subsequent discussion were deliberately focused on the bacterial groups most frequently identified in the included studies. This approach aimed to prioritize the most recurrent and representative evidence within the set of screened articles, allowing for a more consistent and relevant analysis of the microbiological profile observed in the BDL model.

**FIGURE 5:**
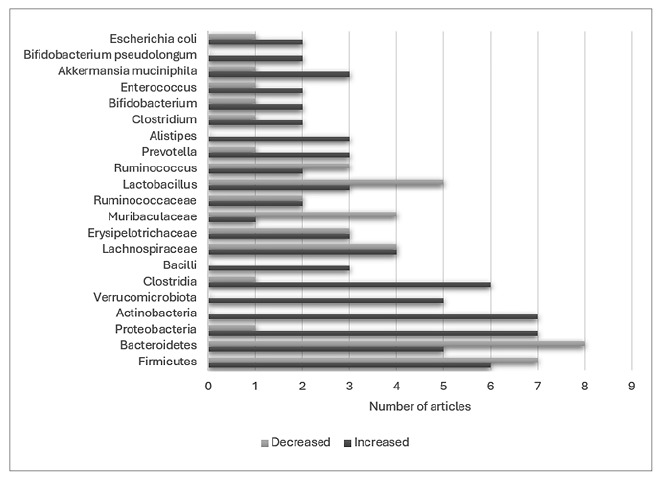
Main Alterations in the Gut Microbiota of Rodents with Cholestasis.

Conflicting results were observed regarding changes in the intestinal microbiota composition in rodents subjected to BDL-induced cholestatic liver disease compared to healthy animals (Figure 5 and SUPPLEMENTARY TABLE S1). Moreover, not all studies provided complete data on diversity indices or taxonomic classifications at the phylum, family, class, genus, and species levels.

**TABLE S1:** Main alterations in the gut microbiota of rodents with cholestasis.

Refs.	Alterations in the Intestinal Microbiota
^1^	**Increased**: Firmicutes, Ruminococcus
	**Decreased:** Bacteroidetes, Lactobacillus
^4^	**Increased:** Clostridia
	**Decreased:** Bacteroidetes
^5^	**Increased:** Bacteroidetes, Actinobacteria,
	Proteobacteria, Clostridium, Bifidobacterium,
	**Decreased:** Ruminococcus, Lachnospiraceae
^6^	**Decreased:** Bacteroidetes
^10^	**Increased:** Firmicutes, Clostridia,
	Lachnospiraceae, Akkermansia muciniphila
^11^	**Increased:** Bacteroidetes, Prevotella
	**Decreased:** Firmicutes, Lactobacillus
^25^	**Increased:** Bifidobacterium
^26^	**Increased:** Firmicutes, Proteobacteria,
	Verrucomicrobiota, Actinobacteria
	**Decreased:** Bacteroidetes, Erysipelotrichaceae, Ruminococcaceae
^30^	**Increased:** Firmicutes, Lachnospiraceae,
	Prevotella, Bacilli, Clostridia, Erysipelotrichaceae
^31^	**Increased:** Proteobacteria, Verrucomicrobiota
	**Decreased:** Bacteroidetes, Firmicutes
^36^	**Increased:** Actinobacteria, Clostridia
	**Decreased:** Firmicutes, Bacteroidetes, Proteobacteria, Lachnospiraceae, Enterococcus, Ruminococcus
^38^	**Increased:** Bacteroidetes, Actinobacteria, Proteobacteria, Clostridium
	**Decreased:** Firmicutes, Lactobacillus, Prevotella, Ruminococcus
^42^	**Increased:** Verrucomicrobiota, Alistipes, Akkermansia muciniphila, Bifidobacterium pseudolongum
	**Decreased:** Lachnospiraceae, Muribaculaceae
^44^	**Increased:** Escherichia coli
	**Decreased:** Bifidobacterium, Lactobacillus
^50^	**Increased:** Bacteroidetes, Proteobacteria, Actinobacteria, Clostridia, Lactobacillus,
	**Decreased:** Firmicutes, Ruminococcaceae, Erysipelotrichaceae, Clostridium, Escherichia coli, Akkermansia muciniphila
^51^	**Increased:** Enterococcus, Bifidobacterium
	**Decreased:** Bacteroidetes
^52^	**Increased:** Enterococcus, Prevotella, *Escherichia coli*
^53^	**Increased:** Firm icutes, Verrucomicrobiota, Bacilli, Lactobacillus, Akkermansia muciniphila, Bifidobacterium pseudolongum
	**Decreased:** Bacteroidetes, Clostridia
^54^	**Increased:** Actinobacteria, Proteobacteria, Bifidobacterium, Enterococcus
	**Decreased:** Firmicutes, Bacteroidetes, Lactobacillus
^55^	**Increased:** *Ruminococcus*
^56^	**Increased:** *Bacteroidetes, Proteobacteria, Verrucomicrobiota, Alistipes*
	**Decreased:** *Firmicutes, Lactobacillus*
^57^	**Increased:** Firmicutes, Clostridia, Bacilli, Lachnospiraceae, Lactobacillus, Alistipes
	**Decreased:** *Bacteroidetes, Muribaculaceae, Erysipelotrichaceae*

### Alterations in alpha and beta diversity indices

Regarding alpha diversity, nine studies reported a reduction in intestinal microbiota diversity indices in rodents subjected to the BDL model compared to control groups. This reduction suggests a loss in microbial richness and/or evenness, possibly due to cholestasis-induced alterations in bile secretion and intestinal barrier dysfunction. Under physiological conditions, primary bile acids are synthesized in the liver, conjugated, secreted into bile, and released into the small intestine, partially converted into secondary bile acids by microbiota. In cholestasis, this bile flow to the intestine is diminished or blocked, reducing the amount of primary bile acids reaching the intestinal lumen and decreasing microbial diversity^24^. These changes may favor the overgrowth of opportunistic microorganisms at the expense of a more diverse and functional microbiota, thereby contributing to the exacerbation of intestinal dysbiosis commonly observed in this experimental model^25^ ^,^ ^26^.

Conversely, three studies reported increased alpha diversity in the BDL group compared to the sham group, six reported no statistically significant difference, and three did not present alpha diversity data. These discrepancies may be attributed to multiple experimental factors, including species and strain of the animals, post-surgical analysis time, and variations in diet or housing conditions^7^ ^,^ ^23^. From a biological perspective, the hepatic inflammatory response resulting from cholestasis may modulate the gut microbiota in diverse ways, favoring either the proliferation of opportunistic microorganisms or, in specific contexts, an increase in microbial diversity as a response to the altered environment^7^ ^,^ ^27^. Thus, the variability across studies reflects the complexity of the gut-liver axis as well as the sensitivity of the intestinal microbiome to small experimental fluctuations.

All studies included in this review reported statistically significant differences in beta diversity indices between BDL and sham groups. These results indicate that the overall composition of the intestinal microbiota was substantially altered by cholestasis induced via bile duct ligation, reflecting a characteristic dysbiosis pattern of the model. Beta diversity analysis, generally based on metrics such as Bray-Curtis, UniFrac, or Jaccard, revealed clear separation between groups in ordination plots (e.g., PCoA or NMDS), reinforcing the notion that the cholestatic condition consistently and profoundly impacts the structure of the intestinal microbial community.

### Alterations in intestinal microbiota composition at the phylum taxonomic level

At the phylum level, the reviewed studies most frequently reported microbial groups were *Firmicutes*, *Bacteroidetes*, *Proteobacteria*, *Actinobacteria*, and *Verrucomicrobiota*. In animals subjected to BDL-induced hepatic cholestasis, significant variability in the relative abundance of the phyla *Firmicutes* and *Bacteroidetes* was observed. Specifically, *Firmicutes* increased in six studies and decreased in seven, while *Bacteroidetes* increased in five studies and decreased in ten. The reduction in the abundance of these phyla, often accompanied by an increase in *Proteobacteria*, is potentially indicative of intestinal dysbiosis and associated inflammation.

The decline in these phyla may lead to reduced production of short-chain fatty acids (SCFAs) such as propionate and butyrate-metabolites essential for maintaining the integrity of the intestinal epithelial barrier and regulating local immune responses. Impairment of these functions may, in turn, result in increased intestinal permeability, greater risk of microbial translocation, and intensification of hepatic inflammation^28^ ^,^ ^29^.

On the other hand, the increase in these phyla, as reported in some studies, reinforces the notion that microbiota composition under cholestatic conditions responds heterogeneously, reflecting the complexity of interactions among the liver, gut, and microbiota.

In contrast, the phyla *Proteobacteria*, *Actinobacteria*, and *Verrucomicrobiota* showed a more consistent pattern of increased abundance in cholestatic models. *Proteobacteria*, mentioned in eight studies (with only one reporting a decrease), comprises numerous Gram-negative bacteria capable of producing lipopolysaccharides (LPS), which activate receptors such as TLR4, promoting inflammation, Kupffer cell activation and hepatic fibrosis progression^30^.

The phylum *Actinobacteria* also plays a relevant role in maintaining intestinal barrier homeostasis, particularly through SCFA production. The increased abundance of this phylum, reported in six studies, may reflect an adaptive or compensatory response to intestinal dysbiosis and local inflammation to restore homeostasis and reinforce barrier function^28^.

Similarly, the elevation of *Verrucomicrobiota*, cited in five studies, may indicate a compensatory mechanism aimed at restoring intestinal mucosal homeostasis, considering that its primary representative, *Akkermansia muciniphila*, is associated with the strengthening of the intestinal barrier^31^.

### Alterations in intestinal microbiota composition at the class taxonomic level

*Clostridia* and *Bacilli*-belonging to the phylum *Firmicutes*-were the most prominently discussed at the class taxonomic level. The class *Clostridia* was reported in seven studies, with six noting an increase in its relative abundance in BDL-subjected models. Although this class includes potentially pathogenic strains that may contribute to intestinal and hepatic inflammation in dysbiotic contexts, *Clostridia* also comprises a substantial proportion of beneficial commensal bacteria within the gut microbiota^32^.

Many of these microorganisms are SCFA producers, particularly of butyrate, an energy source for enterocytes, strengthens intestinal epithelial barrier integrity, and exerts anti-inflammatory effects. Furthermore, as strict anaerobes, these microbes cannot survive in oxygenated tissues, limiting their capacity for translocation to the liver and other organs, thereby conferring a protective role^33^.

The class Bacilli was reported to increase in three reviewed studies. This class includes genera such as *Lactobacillus*, *Enterococcus*, and *Streptococcus*, whose species play distinct roles in the gastrointestinal tract. Although some strains of *Lactobacillus* exhibit probiotic and anti-inflammatory properties, the widespread expansion of *Bacilli* may reflect microbial imbalance associated with altered bile salt concentrations and changes in the intestinal environment under cholestatic conditions.

Unlike *Clostridia*, many members of the class *Bacilli* are facultative anaerobes, which enhances their ability to translocate to other tissues when the intestinal barrier is weakened or their populations are expanded. This may contribute to extraintestinal inflammatory processes, including liver injury^33^ ^-^ ^35^.

### Alterations in intestinal microbiota composition at the family taxonomic level

Among the most frequently reported bacterial families, *Lachnospiraceae* was mentioned in six studies, with four of them indicating an increase in its abundance. *Erysipelotrichaceae* was referenced in four studies, showing a decrease in three of them. *Muribaculaceae* and *Ruminococcaceae* were each mentioned in two studies, and both showed a reduction in their abundances.

*Lachnospiraceae* and *Ruminococcaceae*, both belonging to the class *Clostridia* (phylum *Firmicutes*), play fundamental roles in the production of short-chain fatty acids (SCFAs), which are essential for maintaining intestinal barrier integrity, modulating immune responses, and controlling inflammation. An increase in the abundance of one of these families in cholestatic models may reflect a functional adaptation of the gut ecosystem to changes in fermentable substrate availability and bile acid composition resulting from hepatic dysfunction^36^.

Moreover, both families are directly involved in bile acid metabolism-an exclusive function of the intestinal microbiota-which includes deconjugation, dehydroxylation, and epimerization reactions. The 7α-dehydroxylation step, responsible for converting primary into secondary bile acids, is particularly restricted to a limited number of anaerobic bacteria in the order *Clostridiales*, especially members of the *Lachnospiraceae* families, highlighting their importance in enterohepatic homeostasis^36^ ^,^ ^37^.

Erysipelotrichaceae is a commensal family that can exert context-dependent beneficial effects, particularly in carbohydrate fermentation. However, its reduction under dysbiotic conditions, such as cholestasis, may disrupt metabolic functions typically supported by this group, including the production of beneficial fermentation byproducts. This disruption may impair energy absorption and compromise mucosal barrier integrity, thereby increasing susceptibility to pathogenic colonization and low-grade inflammation^38^.

The Muribaculaceae family has a high capacity to metabolize complex polysaccharides, both endogenous (e.g., mucin-derived glycans) and exogenous (e.g., dietary fibers), and is also involved in SCFA production, particularly propionate. Therefore, the reduction in its relative abundance, observed in most of the studies in which it was cited, may reflect a loss of functional microbial diversity, leading to impaired fiber fermentation and reduced production of beneficial metabolites essential for intestinal homeostasis^39^.

### Alterations in intestinal microbiota composition at the genus taxonomic level

At the genus level, *Lactobacillus* was the most frequently cited, with six studies reporting its reduction and three reporting its increase. *Ruminococcus* was mentioned in five studies, most of which associated it with decreased abundance. *Prevotella* and *Alistipes* were frequently reported to increase following cholestasis induction. *Clostridium* was reported to be increased in two of the three studies that mentioned it. In addition, the genera *Bifidobacterium* and *Enterococcus* were found to be elevated in three of the four studies in which they were mentioned.

Reductions in *Lactobacillus* and *Ruminococcus* are particularly relevant, given their beneficial roles in intestinal health. *Lactobacillus* maintains the epithelial barrier, immune modulation, and inhibition of pathogenic microorganisms, while *Ruminococcus* is involved in butyrate production, a short-chain fatty acid with important anti-inflammatory effects. The loss of these genera may impair barrier function and favor bacterial translocation^1^ ^,^ ^38^.

Conversely, increased genera such as *Prevotella*, *Alistipes*, *Clostridium*, and *Enterococcus* may indicate a dysbiotic microbial profile. Although *Prevotella* is considered a commensal genus under physiological conditions, its excessive expansion has been associated with the degradation of the mucin layer, compromising the integrity of the intestinal barrier and triggering host inflammatory responses. Additionally, species of this genus are involved in the fermentation of dietary polysaccharides, primarily producing acetate, a short-chain fatty acid that, when imbalanced with other metabolites, may contribute to a pro-inflammatory intestinal environment^38^ ^,^ ^25^.

*Clostridium* and *Enterococcus* genera include potentially toxigenic species whose increase may contribute to liver injury and intestinal barrier disruption. Loss of barrier integrity facilitates the translocation of bacteria and their microbial products, promoting immune activation and exacerbating hepatic inflammation^7^ ^,^ ^36^ ^,^ ^40^.

*Bifidobacterium*, from the phylum *Actinobacteria*, and *Alistipes*, from the phylum *Bacteroidetes*, are recognized for their ability to produce SCFAs, particularly acetate and propionate. These metabolites play essential roles in maintaining intestinal barrier homeostasis and protecting against enteric pathogens such as *Escherichia coli* and *Shigella spp*. The increase in their abundance may represent an adaptive response to intestinal inflammation, reflecting a compensatory mechanism in the face of microbial imbalance^28^ ^,^ ^41^.

### Alterations in intestinal microbiota composition at the species taxonomic level

*Akkermansia muciniphila* was the most frequently reported at the species level, with three studies indicating its increased abundance and only one reporting a reduction. *Bifidobacterium pseudolongum* was cited in two studies; both reported an increase, while *Escherichia coli* was mentioned in three studies, with two indicating its expansion.

*Akkermansia muciniphila*, the sole representative of the phylum *Verrucomicrobiota*, metabolizes mucosal sugars and plays a key role in maintaining the intestinal barrier. Although often associated with beneficial metabolic effects, under dysbiotic conditions it may intensify mucin degradation and compromise barrier integrity, thereby contributing to hepatic inflammation. Its proliferation-potentially related both to protective compensatory mechanisms and to the exacerbated catabolism of mucin in response to inflammatory stress-reflects a still controversial role in the context of cholestasis^31^ ^,^ ^42^.

Similarly, *B. pseudolongum*, traditionally recognized as a probiotic species, may expand as a compensatory response to the altered intestinal ecosystem, although its role in cholestasis remains to be fully elucidated^43^.

The increased abundance of potentially pathogenic bacteria, such as *Escherichia coli*, frequently reported under cholestatic conditions, is strongly associated with reduced bile acid concentrations in the intestinal lumen. These compounds act as natural antimicrobial agents, and their reduction favors the proliferation of opportunistic microorganisms^44^. *E. coli*, a Gram-negative bacterium, possesses LPS in its outer membrane that can activate Toll-like receptor 4 (TLR4), triggering inflammatory cascades involving Kupffer cell activation, hepatic inflammation, and fibrogenesis^45^. Furthermore, the proliferation of such bacteria may compromise epithelial barrier integrity, promoting bacterial translocation and aggravating the systemic inflammatory process^46^.

These findings demonstrate that BDL-induced cholestasis promotes the reshaping of the intestinal microbiota, potentially reducing protective bacterial groups and expanding potentially pathogenic microorganisms, thereby exacerbating hepatic inflammation and intestinal barrier dysfunction.

### Microbiota as an integrative axis in gastrointestinal diseases

Beyond the context of cholestasis, intestinal dysbiosis has been widely implicated in other gastrointestinal diseases, such as irritable bowel syndrome, food intolerance, and non-celiac gluten sensitivity. The literature has demonstrated a growing clinical and pathophysiological intersection between irritable bowel syndrome and non-celiac gluten sensitivity, suggesting that the intestinal microbiota plays a central role in the integration between mucosal immunity alterations, visceral hypersensitivity mechanisms, and symptomatic responses triggered by dietary components such as gluten and FODMAPs. In this scenario, a shared pathophysiological model emerges, supported by the gut-microbiota-nervous system axis, in which the microbiota acts as a determining modulator of immune, metabolic, and neurogastrointestinal interactions^47^ ^-^ ^49^.

Although these findings broaden our understanding of the interfaces between different gastrointestinal disorders and reinforce the therapeutic potential of microbial modulation, it is important to note that, in the context of BDL-induced cholestasis, most of the evidence still derives from experimental studies. Thus, extrapolating these results to humans should be done with caution, and well-designed clinical studies are needed to confirm the safety, efficacy, and applicability of these approaches in clinical practice.

## CONCLUSION

The variability observed among the studies analyzed in this review indicates that the composition of the intestinal microbiota under cholestatic conditions responds heterogeneously, reflecting the complexity of the gut-liver axis. Overall, the gathered evidence demonstrates that cholestasis is associated with significant alterations in the composition and functionality of the intestinal microbiota, characterized by dysbiosis, reduced microbial diversity, and the expansion of commensal and potentially pathogenic genera.

These changes may compromise the integrity of the intestinal barrier, favor bacterial translocation, and exacerbate hepatic inflammation. On the other hand, the increased abundance of certain microorganisms-such as *Akkermansia muciniphila*, *Bifidobacterium*, and *Alistipes*-may reflect adaptive responses to intestinal imbalance, potentially playing protective or compensatory roles.

Taken together, the evidence analyzed in this review demonstrates the complexity of microbiota alterations in experimental cholestasis induced by BDL and reinforces the central role of the gut-liver axis in disease progression. Beyond taxonomic changes, understanding the functional and mechanistic basis of these alterations represents an essential step toward advancing knowledge in this field. Future investigations integrating experimental, translational, and clinical approaches will be fundamental to consolidating the therapeutic applicability of strategies based on microbiota modulation.

## Data Availability

data in article: the research data are presented within the article itself (available in sections results and discussion)
